# Impact of stem profile on the revisability and the need for osteotomy in well-fixed cemented revision total knee arthroplasty implants

**DOI:** 10.1007/s00402-022-04559-2

**Published:** 2022-08-05

**Authors:** Alexander Maslaris, Georgios Spyrou, Carsten Schoeneberg, Mustafa Citak, Georg Matziolis

**Affiliations:** 1 Department of Orthopaedics, Waldkliniken Eisenberg, Chair of the Jena University Hospital, Klosterlausnitzer Str. 81, 07607 Eisenberg, Germany; 2 Department of Orthopedics and Trauma Surgery, Alfried-Krupp Hospital Essen, Alfried-Krupp-Straße 21, 45131 Essen, Germany; 3 Department of Orthopaedic Surgery, HELIOS ENDO-Clinic Hamburg, Holstenstraße, 222767 Hamburg, Germany

**Keywords:** Stem design, Conical stems, Well-fixed cemented stems, Stem removal, Revision total knee arthroplasty, Osteotomy, Revisability

## Abstract

**Introduction:**

While re-revision total knee arthroplasty (ReRTKA) steadily increases, the ease and bone-sparing removal of RTKA implants is gaining more and more in importance. Biomechanical data suggest that cemented conical stems can be removed significantly easier than cylindrical stems. However, no clinical evidence exists supporting this observation. Aim of this study was to compare the revisability and need for osteotomy (OT) between removals of well-fixed cemented conical vs. cylindrical RTKA stems.

**Materials and methods:**

55 removals of well-fixed full-cemented RTKA stems (29 knees) performed between 2016 and 2018 were retrospectively analyzed. Main outcome variables were: bone loss, fractures, osteotomy incidence, surgery duration, early postoperative complications (EPC), hemoglobin drop and blood transfusion. SPSS was used for the statistical analysis.

**Results:**

44.8% were conical, 48.3% cylindrical, and 6.9% combined stem designs. Causes for re-revision were PJI (75.9%), malposition (17.2%) and persistent pain (6.9%). 10 stem removals (18.2%) required an OT (four femoral, six tibial): eight stems (14.5%) had cylindrical and two (3.6%) conical designs (*P* = 0.041). Fractures were noted solely in removals without OT (11.1% vs. 0%,). There was a tendency to more bone loss in cylindrical stem revisions (53.8% vs. 32%, *P* = 0.24). A longer overall surgery time was observed in revisions of cylindrical stems (+ 37 min, *P* = 0.05). There was higher hemoglobin drop and need for blood transfusion in revisions of cylindrical stems or after OT but without reaching statistical significance. The EPC rates were slightly higher in ReRTKA on cylindrical stems (*P* = 0.28).

**Conclusion:**

Well-fixed cemented conical stems may be revision friendlier with less demands on OT and shorter overall surgery time than cemented cylindrical stems.

## Introduction

With small regional differences between national registers [1], revision total knee arthroplasty (RTKA) is steadily increasing worldwide [1–6]. RTKA is associated with increased surgery duration, length of stay, complication rates, and together with the use of more expensive revision implants its impact on health economy remains considerably higher than that of the primary TKA [7].

Along with that, re-revisions are becoming likewise more common in the daily practice of orthopedic centers. With a 78–96% of 10-year survivals [8–11] and failure rates of 10–30%, depending on the background of first revision [9–17], no satisfying improvement in the outcomes of RTKA can be observed during the last decades [8].

Leading indication for re-revision still remains the periprosthetic joint infection (PJI) (30–46%). Further common causes for re-revision are stiffness (10.5–23%), instability (2.9–26%) and other reasons like implant malpositioning, on-going pain and extensor mechanism problems of the knee [8, 9, 11–13, 16]. In all these cases, well-fixed implants with stem extensions that may require removal often coexist. This makes RTKA exchange procedures even more challenging, especially in cases of PJI with cemented stem fixation, where all hardware and cement remnants must be completely removed. Explantations of well-fixed implants are often associated with increased bone loss and intraoperative risk for fractures, prolonged surgery duration and a higher incidence of early perioperative complications.

The complexity of joint reconstruction and relative RTKA outcomes are strongly determined by the severity of bone damage caused during the initial implant removal.

The difficulty in removing well-fixed stem extensions depends not only on the fixation technique (cemented vs. cementless) or bone quality but also on the design of the stem (diameter, length, offset, surface roughness, cross-section and the conicity of the longitudinal profile). While cementless and hybrid fixation techniques are commonly combined with cylindrical stems [18–21], cemented fixation has been used for both, conical [22, 23] and cylindrical stems [24–27]. Biomechanical studies have shown that cemented conical stems can be removed easier than cylindrical designs. Although there are different implant design properties that might have an influence on the difficulty of detaching a stem from its surrounding cement mantle, biomechanical studies have shown that the conicity of the longitudinal stem profile (conical angle and proportion in relation to stem length) is the most strongest predictor [28, 29]. However, no clinical evidence exists until now supporting the above biomechanical observations.

As orthopedic surgeons are increasingly confronted with this challenging entity that can present as an emergency at any time, there is an urge for improved treatment strategies and in-depth understudying of implant-associated influences in the revisability.

Purpose of the current study was to analyze and compare revisions of well-fixed cemented RTKA conical versus cylindrical stem extensions and their need for osteotomy to facilitate implant removal.

## Materials and methods

After ethical approval was granted from the local Institutional Review Board, a retrospective single-center cohort study was conducted in an orthopedic university hospital.

All re-revisions of well-fixed cemented RTKA stems between 4/2016 and 2/2018 were identified and analyzed:

The RTKA stems were divided according to their macroscopical design in: (1) conical (*Co*) (2) cylindrical (*Cy*) and (3) combination profiles (*Combi*: conical-cylindrical or cylindrical-conical).

Revisions or re-revisions dealing with: (1) cementless stem extensions, (2) single liner exchange, (3) isolated patella treatment, (4) solely soft tissue management, (5) removal of primary TKA implants that did not possess a stem extension, (6) partial component removal other than the RTKA stems, (7) spacer exchange, and (8) removals of RTKA implants with metaphyseal sleeve or cone, (9) re-revisions due to periprosthetic fractures or (10) due to implant loosening (septic or aseptic) and finally (11) reimplantations were excluded from the study.

Radiographic criteria for Implant loosening were: (1) any radiolucent line (RLL) of ≥ 2 mm width at any zone according to the Knee Society Roentgenographic Evaluation System [30] at the implant-cement (ICI) or the cement–bone interface (CBI), (2) component migration, (3) subsidence of components due to bone collapse, (4) cement fractures and (5) changes in the degree of knee angulation on weight bearing X-rays [31, 32]. As it is unclear in what extend a RLL of 1–2 mm could impact the explantability of otherwise well-fixed cemented stems, we included only homogeneous cases with absence of any RLL (at any zone) at both interfaces. Implant loosening was then confirmed intraoperatively and documented on the medical reports, respectively.

Demographic data of the patients were collected using the institutional database (Table 1). The primary outcomes of interest (POI) were: (1) Bone loss and (2) fractures induced during implant removal, (3) osteotomies (OT) performed to facilitate removal of well-fixed stem extensions, (4) surgery duration, (5) hemoglobin (Hb) drop and incidence of packed red cell transfusions (PRCT) required, (6) early postoperative surgical site complications (EPC) requiring reoperation, and separately, the incidence of (7) deep vein thrombosis (DVT) and (8) pulmonary embolism (PE) that occurred during hospital stay.

**Table 1:** Removal of well-fixed cemented RTKA implants: demographics and epidemiological characteristics between conical, cylindrical, and combined stem profiles

	Conical (Co)	Cylindrical (Cy)	Combination (Combi)	All	Sign Co vs. Cy
RTKA implants included	13 (44.8%)	14 (48.3%)	2 (6.9%)	29 (100%)	
Components included (f & t)	26	28	4	58	
Components removed (f & t)	25 (96.2%)	26 (92.9%)	4 (100%)	55 (94.8%)	
Age	67.5 ± 9.6	70.1 ± 8.9	40.5 ± 31.8	66.9 ± 13	0.47
Gender (females)	8 (61.5%)	6 (42.9%)	1 (50%)	15 (51.7%)	0.50
Revision indication					
PJI	11 (84.6%)	0.3410 (71.4%)	1 (50%)	22 (75.9%)	0.68
Malposition	2 (15.4%)	3 (21.4%)	0 (50%)	5 (17.2%)	0.69
Persistent knee pain	0 (0%)	1 (7.1%)	1 (50%)	2 (6.9%)	0.34
Previous RTKAs					
1R	2 (15.4%)	1 (7.1%)	0 (0%)	3 (10.3%)	0.49
2R	2 (15.4%)	2 (14.3%)	1 (50%)	5 (17.2%)	0.76
≥ 3R	9 (69.2%)	11 (78.6%)	1 (50%)	21 (72.4%)	0.31

Co, conical stem design; Cy, cylindrical stem design; Combi,  combined conical-cylindrical stem design; RTKA,  revision total knee arthroplasty; f, femoral; t, tibial; PJI, periprosthetic Joint Infection; 1R, one previous revision; 2R, two previous revisions; 3R,three previous revisions; > 3R,  more than three previous revisions; 1 s, one-stage RTKA; 2s,  two-stage RTKA

*Significant at the 0.05 level

Bone defects as detected after implant removal and cement plug extraction were classified according to the Anderson Orthopaedic Research Institute (AORI) classification system [33]. Defects with at least metaphyseal damages AORI ≥ II were considered for evaluation and their incidences were noted. Furthermore, iatrogenic fractures that were associated with the extraction of the stems were also noted. The data were extracted from the surgical reports and confirmed through intraoperative and/or postoperative X-rays and in some cases also computed tomography.

The Hb drop was calculated from the difference between the direct preoperative value and at 5th day after surgery. Cases that received any PRCT during this period were excluded. A restrictive transfusion protocol was used with transfusion trigger Hb < 7 g/dL or Hb < 8 g/dL when clinically relevant or by patient with related cardiovascular risk factors [34].

The POIs were then analyzed and compared: (1) between conical vs. cylindrical stem designs and (2) between RTKA removals with OT vs. without OT.

For the statistical analysis, the software IBM SPSS Statistics (Version 25) was used. For comparisons between non-normally distributed variables the non-parametric tests Kruskal–Wallis or the Mann–Whitney U test were used. To compare differences of frequencies, the Chi-Square test was used. To determine the sample sizes required to reach significance in the comparison of the osteotomy rates between *Co* and *Cy* stem designs, a power analysis (G*Power 3.1) was performed using the Wilcoxon–Mann–Whitney test (two groups) with an effect size = 0.8, given an *α* = 0.05 and a power 0.8. A total of 42 components (21 for each group) would be necessary for this purpose.

### Surgical procedure

The index revisions, equally divided, were performed by three high-volume experienced arthroplasty surgeons with similar skill levels. The procedure of implant removals was standardized with equal sequencies in most cases: to disrupt the implant–cement interface thin sharp osteotomes in different widths (rigid or flexible), power saws with small oscillating blades and a Gigli saw especially for the femoral component were commonly used. For the disimpaction of the components, punches, hooks and universal extraction, devises for axial blows were available. If no implant movement could be detected after the first blows, further attempts were made to completely detach the implant–cement interface before continuing with additional disimpactions. This procedure was repeated until complete implant removal could be achieved.

Early decision for extended osteotomy was made to avoid severe and uncontrolled bone damages through frustrated attempts if the stem was still unable to be detached after some repetitions (approximately 5–10 min) of intramedullary trials, especially in cases of: (1) severe osteopenic bone, (2) cemented offset-stems, (3) cemented stems with a rough surface, (4) cemented long voluminous stems and (5) septic revisions with well-fixed stems and cement plugs deep in the diaphysis requiring complete removal.

**Fig. 1:**
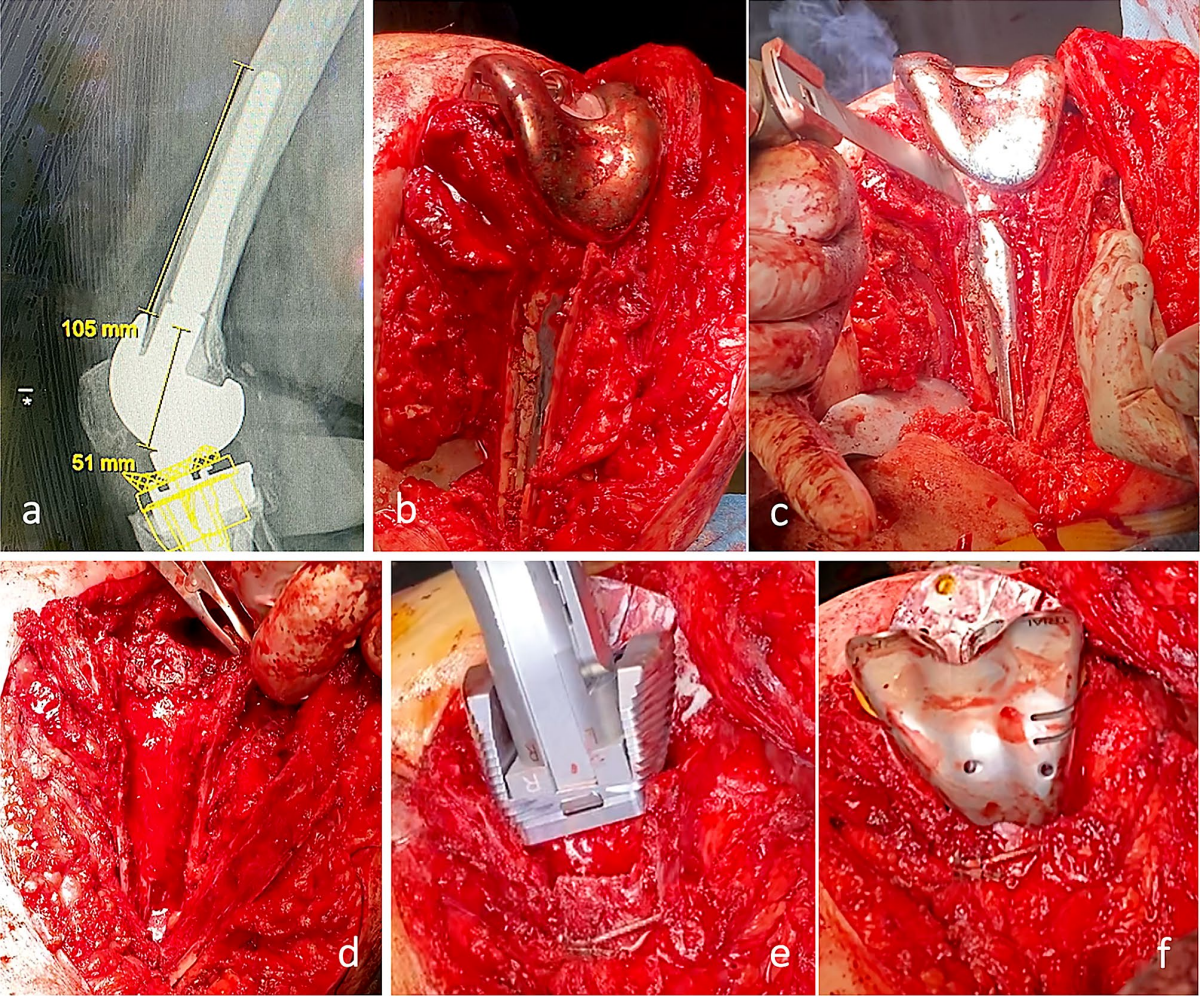
**a**–**f** Example of a removal of a femoral well-fixed cemented offset stem-extension using an osteotomy (**a**–**d**). After cerclage wiring of the OT fragment a subsequent trialing with a metaphyseal cone (**e**) and augments (**f**) was performed to reconstruct the joint

The extended OT was as long and wide as needed to be able to access the implant–cement interface of the entire stem length and the cement plug. The length of the OT was usually preoperatively templated to reach the tip of the stem or a little bit above it to allow a sufficient bridging of the OT with a long stem afterwards during the reimplantation (Fig. 1a–f). On the tibial side, an extended tibial tubercle osteotomy was performed medially leaving the anterolateral tibial muscles attached and an intact soft tissue hinge on the lateral cut of the OT flap. For the medial and distal transverse, OT cuts an oscillating saw with a thin blade was used, whereas the lateral side was opened in an inside-out manner passing from medial to lateral in front of the stem, as described by Massin et al. [35]. On the femoral side, an anteromedial osteotomy below vastus medialis to preserve the extensor mechanism was performed leaving the laterally attached soft tissues and the lateral intermuscular septum intact to preserve vascularization. The osteotomies were performed in the same fashion as described above [35]. To reduce the risk of iatrogenic fractures on the dorsal diaphysis, drill holes were made on the OT path before proceeding with the cuts. The OT were afterwards refixed with either cerclage wires that do not endanger the periosteal vascularization or screws (mostly tibial).

## Results

From a total of 58 RTKA components (29 joints) that were included in the study, 55 (94.8%) well-fixed cemented stem extensions were explanted (Table 1). 25 (45.5%) of the explanted stems had *Co* profile, 26 (47.3%) *Cy* profile and 4 (7.3%) a *Combi* stem design. 27 (49%) were femoral components and 28 (51%) tibial components.

75.9% of the revisions were indicated due to PJI with otherwise no evidence of implant loosening. Other causes were implant malposition (17.2%) and persistent knee pain (6.9%). 72.4% were complex RTKA cases with ≥ 3 previous revisions. The mean patient age was 66.9 ± 13 and 51.7% were females.

The overall incidence of bone loss AORI ≥ II and intraoperative fractures induced during implant removal are shown in Table 2. In ten cases (18.2%), an extended osteotomy was performed (four femoral, six tibial) to facilitate complete removal of the well-fixed cemented stem. The mean surgery time was 140.9 ± 40.3 min. There was a mean Hb drop of 2.3 ± 2.0 g/dL observed. A total of 26 packed red cell transfusions were given in 31% of the cases. 2 (6.9%) DVT and 1 (3.4%) PE occurred during the postoperative hospital stay. 9 (31%) early postoperative surgical site complications (one hematoma and eight wound infections) required reoperation (Table 4).

**Table 2:** Revisability analysis between different stem profiles during removals of well-fixed cemented RTKA stems

	Conical (Co)	Cylindrical (Cy)	Combination (Combi)	All	Sign Co vs. Cy
Components removed					
All	25 (45.5%)	26 (47.3%)	4 (7.2%)	55 (100%)	0.90
f	13	12	2	27	
t	12	14	2	28	
Bone loss					
All	8 (32%)	14 (53.8%)	0 (0%)	22 (40%)	0.24
f	*4*	*6*	*0*	*10*	
t	*4*	*8*	*0*	*12*	
Fractures					
All	2 (8%)	2 (7.7%)	1 (25%)	5 (9.1%)	0.97
f	*1*	*1*	*1*	*3*	
t	*1*	*1*	*0*	*2*	
Osteotomy					
All	2 (8%)	8 (30.8%)	0 (0%)	10 (18.2%)	0.04*
f	*1*	*3*	*0*	*4*	
t	*1*	*5*	*0*	*6*	
Hb drop *(g/dL)*	2.1 ± 1.8	2.9 ± 2.3	1.9 ± 2.3	2.3 ± 2.0	0.57
PRCT (*%* of transfused patients)	18%	42%	0	30%	0.22
Surgery duration (*min*.)	125.7 ± 30	162.7 ± 44	127 ± 32	140.9 ± 40.3	0.05*

Co, conical stem design; Cy, cylindrical stem design; Combi,  combined conical-cylindrical stem design; f,  femoral; t,  tibial; Hb,  hemoglobin; PRCT,  packed red cell transfusion

*Significant at the 0.05 level

A comperative group analysis (*Co* vs. *Cy* Stems; *With* vs. *Without OT*) yielded following resutls:

### Co vs. Cy stems

**Fig. 2:**
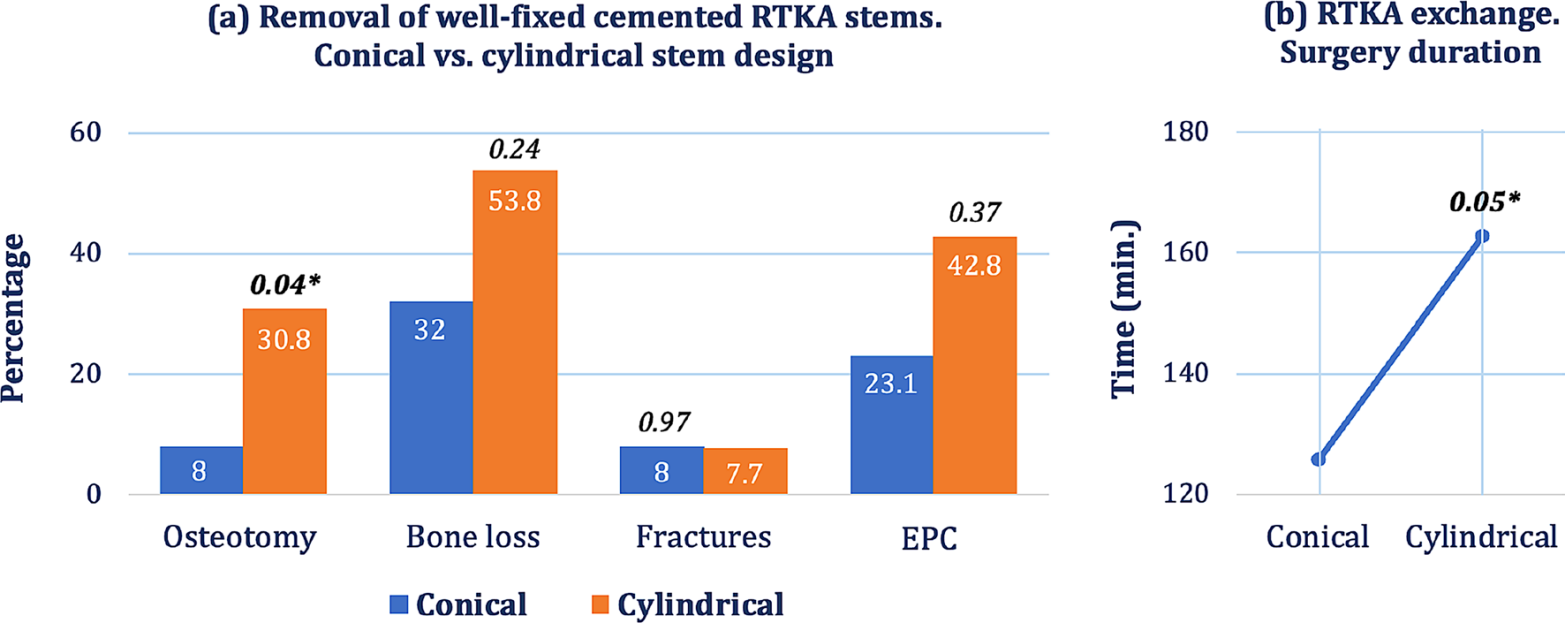
**a** Comparison of the revisability between well-fixed cemented conical stems vs. well-fixed cemented cylindrical stems. **b** A tendency to longer overall mean surgery time was observed in revisions of cylindrical stems. * Significance at the 0.05 level

Cylindrical stems were associated with a significantly higher incidence of OT compared to the conical stems (30.8% vs. 8%, *P* = 0.04). Tibial osteotomies were more frequent than femoral osteotomies (6 vs. 4) (Table 2, Fig. 2a, b).

A slightly higher tendency for bone loss was observed in cylindrical stems than the conical stems but with no statistical significance (53.8% vs. 32%, *P* = 0.24). More bone losses occoured on the tibial side than the femoral side (12 vs. 10). No difference in intraoperative fractures was found between the two stem designs (8% vs. 7.7%, *P* = 0.97).

The removal of well-fixed cylindrical stems was associated with a significantly higher overall surgery duration with a mean difference of + 37 min to the revisions of conical stems (*P* = 0.05). Respectively, there was a higher level of Hb drop in *Cy* stems than *Co* stem revisions (2.9 ± 2.3 g/dL vs. 2.1 ± 1.8 g/dL, *P* = 0.57) with more patients requiring PRCT (41.6% vs. 18.2%, *P* = 0.22).

*Cy* stems had a higher incidence of EPC [42.8% with five wound infections and one hematoma) compared to *Co* stem revisions (23.1% EPC including three wound infections) but with no statistical significance (*P* = 0.642). 1 DVT and one PE occurred in the *Co* groups, whereas one DVT and no PE in the *Cy* group (Table 4).

### With vs. without OT

The overall incidence of OT during the explantations of well-fixed cemented RTKA implants was 18.2%: ten osteotomies were performed in nine knees, involving eight cylindrical stems and two conical stems, four on the femoral side and six on the tibial side (Table 3). The prevalence of cylindrical stem removals in the RTKA group with OT was significantly higher compared to the group without OT (80% vs. 40%, respectively, *P* = 0.022).

**Table 3:** Revisability analysis between removals of well-fixed cemented RTKA stems with OT vs. without OT

	Without OT (øOT)	With OT (OT)	All	Sign øOT vs. OT
Components removed				
All	45 (81.8%)	10 (18.2%)	55 (100%)	0.000**
f	23	4	27	
t	22	6	28	
Bone loss				
All	19 (37.8%)	3 (30%)	22 (40%)	0.48
f	8	2	10	
t	11	1	12	
Fractures				
All	5 (11.1%)	0 (0%)	5 (9.1%)	0.27
f	3	0	3	
t	2	0	2	
Stem design				
Co				
All	23 (51.1%)	2 (20%)	25 (45.5%)	0.07
f	1	1	13	
t	11	1	12	
Cy				
All	18 (40%)	8 (80%)	26 (47.3%)	0.02*
f	9	3	12	
t	9	5	14	
Combi				
All	4 (8.9%)	0 (0%)	4 (7.3%)	0.33
f	2	0	2	
t	2	0	2	
Hb drop (g/dL)	2.3 ± 1.9	2.4 ± 1.9	2.3 ± 2.0	0.97
PRCT (% of transfused patients)	29%	40%	31%	0.62
Surgery duration (min)	141.9 ± 41	131.5 ± 50	140.9 ± 40	0.82

OT, osteotomy; f,  femoral; t,  tibial; Co,  conical stem design; Cy,  cylindrical stem design; Combi,  combined conical-cylindrical stem design; Hb, hemoglobin; PRCT, packed red cell transfusion

**Significance at the 0.01 level

*Significance at the 0.05 level

**Fig. 3:**
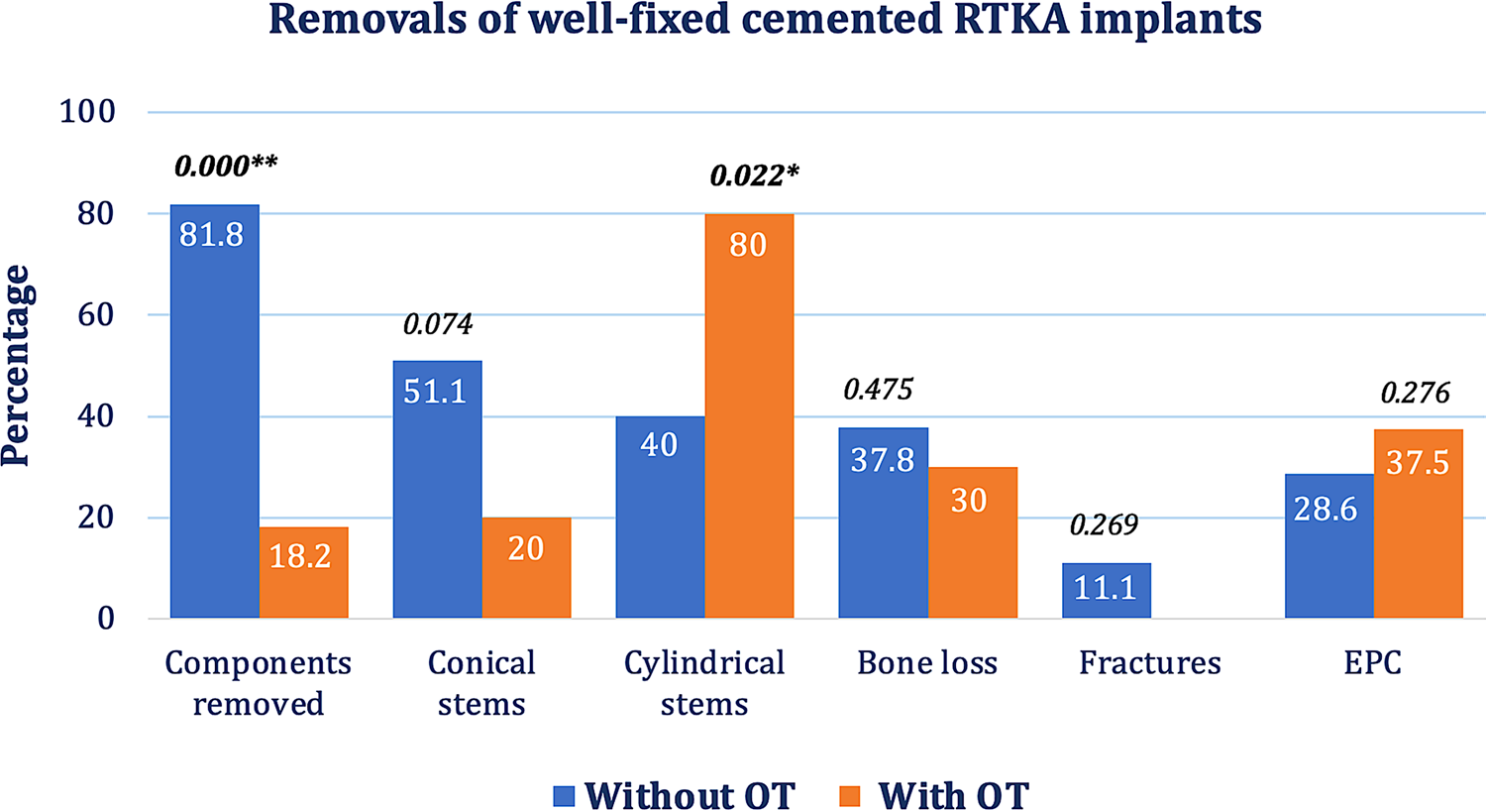
Comparison of well-fixed cemented RTKA implant removals that were done without OT and with OT. The numbers above the columns represent *P* values. ** Significance at the 0.01 level. * Significance at the 0.05 level

There was a slight tendency towards more bone loss in the no-OT group over the OT group (37.8% vs. 30%) but without reaching statistical significance (*P* = 0.48). No intraoperative fractures were observed in the OT group as opposed to the incidence of 11.1% in the no-OT group (*P* = 0.27) (Table 3, Fig. 3).

The surgery duration showed small differences between both groups (131.5 ± 50 vs. 141 ± 41, P = 0.82). While the PRCT-free mean Hb drop was similar for both groups OT vs. no-OT (2.4 ± 1.9 g/dL vs. 2.26 ± 1.9 d/dL, respectively, *P* = 0.97), the incidence of required packed red cell transfusions was 40% for the OT group and 29% for the no-OT group (*P* = 0.618).

There were no DVT or PE in the OT group and the incidence of EPCs was 23.1% with three reinfections. The group without OT developed two (9.5%) DVT, one (4.8%) PE and in 28.6% EPCs including five wound infections (23.8%) and one hematoma (4.8%). However, the incidence of EPC between the two groups showed no statistically significant difference (*P* = 0.276) (Table 4).

**Table 4:** Complication analysis between (a) RTKA stem designs (Co vs. Cy) and (b) implant removals with OT vs. without OT

Comparisons	DVT	PE	EPC
Stem design			
Co	1 (7.7%)	1 (7.7%)	3 (23.1%) wound infection
Cy	1 (7.1%)	0	1 (7.1%) hematoma 5 (35.7%) wound infection
Implant removal			
With OT	0	0	3 (37.5%) wound infection
Without OT	2 (9.5%)	1 (4.8%)	1 (4.8%) hematoma 5 (23.8%) wound infection
All	2 (6.9%)	1 (3.4%)	9 (31%)

DVT, deep vein thrombosis; PE,  pulmonary embolism; EPC,  early postoperative complications; Co,  conical stem design; Cy,  cylindrical stem design; With OT,  RTKA with osteotomy; Without OT,  RTKA without osteotomy

## Discussion

Main result of the current study is the clinical relevance of stem profile and its impact on the revisability of well-fixed cemented RTKA stem extensions during their removal. While conical stems could be removed relatively easily without requiring any extended osteotomy in most cases, cylindrical stem profiles were associated with a significantly higher incidence of OT to enable their extraction (8% vs. 30.8%, *P* = 0.04), (Table 2). 80% of the osteotomies observed in the study were performed during cylindrical stem revisions (*P* = 0.02), (Table 3).

Furthermore, revisions of well-fixed cemented cylindrical stems revealed a tendency to more bone loss (52.8% vs. 32%, *P* = 0.24), a higher PRCT-free hemoglobin drop (2.9 ± 2.3 g/dL vs. 2.1 ± 1.8 g/dL, *P* = 0.57) and an increased incidence of required packed red cell transfusions (41.6% vs. 18.2%, *P* = 0.221) when compared to the conical stem designs. Respectively, a longer surgery duration with a significant mean difference of 37 min (*P* = 0.05) was observed in the revision group of cylindrical stems versus the cemented conical stem revisions. Thus, although it is well known that there are several reasons for iatrogenic bone loss and fractures other than the stem removal during RTKA exchanges [36, 37], it can be postulated that in sum, removals of well-fixed cemented cylindrical RTKA stems are more challenging, hazardous and potentially more invasive when compared to equal cases with conical stem designs.

The findings of this investigation align with the observations published in recent biomechanical studies that could demonstrate that cemented cylindrical stems, independent of other design properties, require the highest extraction energy when compared to conical stem profiles [28, 29].

The incidence of intraoperative fractures observed in this study was 9.1%, all of which occurred during revisions of well-fixed cemented stems that were removed without OT (Table 3, Fig. 3). Sassoon et al. [36] reported in a retrospective study on 894 staged TKA reimplantations for PJI an overall incidence of 2.3% intraoperative fractures, of which 18% occurred during implant extraction. Our cohort included mostly cases of severe complexity with 72.4% carrying ≥ 3 previous revisions, 75.9% being done due to PJI or recurrent PJI, all of which involving well-fixed cemented stem extensions. Thus, the combination of high energy disimpactions for the removal of stable implants and a surrounding poor bone stock often seeing in septic re-revisions can explain the increased fracture incidence observed here.

Therefore, in these challenging re-revision scenarios, an extended osteotomy may be the best solution in order to facilitate sufficient removal of the well-fixed cemented cylindrical stems and the cement plug, which is most important in cases of PJI. Thus, early decision in favor of an OT if indicated may be advantageous minimizing the risk of further iatrogenic and uncontrolled bone damages, and in many cases improving surgery time (Table 3). However, although OT was here not associated with an increased incidence of bone loss or complications when compared with the RTKA removals without OT, it was related with a higher transfusion rate, which reflects to the higher risk of blood loss after osteotomies as reported also in previous studies [35, 38].

Despite all multifarious approaches to improve results, the overall outcome of RTKA mostly due to the high incidence of PJI is generally still far from satisfactory. With failure rates of RTKA ranging between 10 and 30%, no significant tendency of improvement can be confirmed in the past decades [39]. Leading cause for re-revision is still the PJI (30–46%). Further causes for re-revision are also instability (2.9–26%), stiffness (10.5–23%) and other reasons such as malposition, malalignment, on-going pain and extensor mechanism problems [8, 9, 11–13, 16] that very often coexist with well-fixed stem extensions. The greater the damage caused through an RTKA exchange procedure, the more complicated and difficult becomes the joint reconstruction and implant fixation followed. Thus, more focus should be shifted to the revisability and easiness (bone-sparing) of exchange procedures, to insure best possible preconditions for the upcoming revisions [40].

Extraction techniques of well-fixed cemented or uncemented RTKA implants can be challenging and are linked with increased risks for complication [40–47]. Ultrasonic methods [48], pneumatic shock wave technology and intramedullary endoscopy (mostly for hip procedures but also for the knee) [49, 50] customized guides [51], high-powered drills with centralizers [52], and computer assisted freehand navigations [53] are new technologies and methods for implant removal in revision total knee and hip arthroplasties that aim to reduce complication risks and possibly the need for osteotomies. However, due to several limitations and still missing reliable evidence for the superiority of one or another method, these techniques rely mostly on the individual preference of the surgeon and have not gained enough acceptance until now to prevail on the daily praxis in RTKA. Thus, cortical osteotomy still remains a standard approach and a reliable option.

Different osteotomy techniques for removal of femoral stem extensions have been described in the literature: (1) The midline anterior distal femoral osteotomy by Merz et al. [45], (2) the extended femoral osteotomy by Massin et al. [35], which runs anteriorly from medial to lateral under the muscle of vastus medialis to preserve the vascularization of the fragment und (3) the anterolateral oblique distal femoral osteotomy by Fehring et al. [54], which also serves the protection of the suppling periosteum.

Tubercle osteotomies on the tibia for exposure purposes in RTKA have been commonly performed from medial to lateral and pedicled laterally with the anterior tibial muscle and the periosteum to preserve vascularization [46, 47, 55]. Lateral to medial tubercule osteotomies have been also described in combination with a lateral parapatellar TKA approach [56]. However, in difficult RTKA removals of well-fixed stems a more extended osteotomy adjusted on the stem length and the level of the cement plug is required [35, 40]. All osteotomy techniques presuppose a good knowledge of the anatomy of the surrounding periosteal flaps to avoid healing problems [57].

Implant-associated risk factors for difficult exchange procedures of well-fixed RTKA components that may indicate the use of an osteotomy are particularly (1) well-fixed metaphyseal sleeves [42] or (2) trabecular metal-monoblock components [58, 59], (3) offset stem, (4) long stems with large diameter (> 14 mm), (5) curved stem designs and finally, (6) cylindrical stem designs (with low longitudinal conical profile of less than 0,25° angle and < 20% conical proportion [28]. A further indication for an early potential osteotomy is a deep cement penetration at the level of the diaphysis in the presence of an infection.

The current study showed that extended osteotomy for implant removal, especially in cases of well-fixed cylindrical stems was not associated with significant differences in procedure-specific EPC and re-revision rates compared with RTKA removals without OT (Table 4). All EPCs that led to an early re-revision during hospital stay were hematomata and wound reinfections as most patients were complicated septic cases with several comorbidities and previous revisions in their medical history and not complications associated with the osteotomy. This finding corresponds to the existing literature that reveals also satisfying results after OT in septic RTKAs [35, 60]. Therefore, orthopedic surgeons should be encouraged to take early decision for osteotomy.

This is the first study evaluating the clinical relevance of stem design on the revisability and the need of osteotomy during challenging removals of RTKA implants on 29 cases involving 55 explanations of well-fixed cemented stem extensions. Furthermore, to improve the sample homogeneity differentiations and subgroup analyses were conducted.

The limitations of the study include the small sample sizes induced by the subgroup analysis. Another limitation of the study design was its retrospective character and the intraoperative data collection based on surgery reports, which might comprise an interobserver variation and therefore a potential bias. However, all findings were reviewed and verified by X-rays and CT.

The relative inhomogeneity of the samples including cases that ranged from one previous revision to a complex septic RTKA with multiple (< 3) previous revisions presents another limitation. However, with a 74.2% of ReRTKA cases having ≥ 3 revisions, the patient group included in the current study represents mostly the complex RTKA.

The Hb drop values in this study do not mirror the exact estimated blood loss, as we did not consider cases that received PRCT during the evaluation time. Furthermore, Hb drop is multifactorial (age, gender, BMI, hydration status of the patient and examination time) and can individually vary [61].

The mean surgery time as evaluated for all RTKAs revealed an increasing tendency when cylindrical stems were involved. However, this finding must be interpreted with caution as it involved the overall surgery time and not the time required for the removal of the stem alone, which was difficult to detect in a retrospective study setting. Thus, the validity of this information is limited. Nonetheless, our results are consistent to the available literature and reflect our experience, providing some guidance to attending physicians on the proper choice of implants and techniques for a more reproducible and controllable management of RTKA implant revisions.

Further prospective studies with larger series will bring more clarity and transparency to the current topic.

## Conclusion

Improved revisability of RTKA exchange procedures remains a central point of interest. Implants with superior or equal durability and at same time friendlier revisability are preferable. While removal of well-fixed cemented cylindrical RTKA stem extensions can be challenging, conical stems may be revision friendlier with less frequent need for an osteotomy.
